# Assessing a PSP (primary care support programme) from the point of view of the professionals involved: A joint-effort between primary care and psychiatric ward

**DOI:** 10.1192/j.eurpsy.2021.1078

**Published:** 2021-08-13

**Authors:** S.F. Contaldo, D. Carbonell Simeon, B. Rodriguez Ferraz, E. Blanco García, R. Fernandez Vergel, M. Iglesias Gonzalez, M. Rubio Valera, M. Gil Girbau, M.T. Peñarrubia Maria

**Affiliations:** 1 Psychiatry, Parc Sanitari Sant Joan de Deu, Esplugues de Llobregat, Spain; 2 General Practice, ICS, Gavá, Spain; 3 Psychiatry, ICS, Badalona, Spain; 4 Consortium For Biomedical Research In Epidemiology And Public Health Network (ciberesp), Parc Sanitari Sant Joan de Deu, Sant Boi de Llobregat, Spain; 5 Group On Health Technologies And Results In Primary Care And Mental Health (prisma), Fundació Sant Joan de Deu, Sant Boi de Llobregat, Spain

**Keywords:** qualitative study, mental health, Collaborative care

## Abstract

**Introduction:**

The PSP has been implemented in Catalonia in 2006 in an attempt to improve the Primary Care treatment of the most common mental disorders and addictions. It’s based on a collaborative model, made up between Primary Care and Mental Health professionals.

**Objectives:**

To identify the strengths and limitations of the PSP from the perspective of Primary Care and Mental Health professionals.

**Methods:**

Qualitative, exploratory and interpretive study based on Grounded Theory, made between 2018 and 2019 with Primary Care and Mental Health professionals. Group interviews were conducted with triangulated analysis. The study got the approval from the Research Ethics Committee of the Sant Joan de Deu’s foundation.

**Results:**

11 group interviews were conducted in 6 primary care centers and 5 mental health centers in Barcelona. Intrinsic and extrinsic factors impacting the programme functioning were detected. Within the extrinsic factors, elements related to professionals, patients and public health system have been observed. All the professionals agree that the PCSP has a favorable impact on inter-professional relationships and patients, facilitating the management of cases. In contrast the heterogeneity implementation, the lack of training, and the health care burden in is considered to negatively influence an optimal development of the programme. Professionals suggest communication and inter-professional collaboration would be improved by creating more a horizontal structure that eliminates vertical lines of command and disagreements in clinical judgement, thus facilitating shared decisions.

**Conclusions:**

PrimaryCare and MentalHealth professionals value the PSP positively, but conclude there are communication and organizative barriers that should be addressed in order to improve the overall programme’s efficiency.

